# Caregiving Intensity and Depressive Symptoms Among Older Adults After Partners’ Onset of Dementia

**DOI:** 10.1093/geroni/igab046.583

**Published:** 2021-12-17

**Authors:** Melissa Harris, Jinkyung Ha, Geoffrey Hoffman

**Affiliations:** 1 Duke University, Durham, North Carolina, United States; 2 University of Michigan, Ann Arbor, Michigan, United States

## Abstract

Caring for a partner with Alzheimer’s disease or related dementia (ADRD) can create tremendous care burdens. However, the extent to which onset of ADRD in a partner impacts caregiving intensity and emotional health, the relationship of increased care intensity to emotional health, and whether relationships vary across the older adult population, are less clear. We used 9 waves (years 2000-2016) of the nationally representative Health and Retirement Study dataset to examine the number of weekly caregiving hours provided and depressive symptoms for older (ages ≥51) individuals after partners' ADRD onset (measured with the Telephone Cognitive Interview Survey). We compared changes in outcomes from before to after partners' ADRD onset using zero-inflated negative binomial regression models, overall and among sub-populations with potential vulnerability to excess care burdens – women and racial/ethnic minorities. In our sample of 2,186 older Americans with 10,120 unique observations, we observed a 215% increase (p<0.001) in weekly caregiving hours provided and 21% increase (p<0.001) in depressive symptoms reported by older respondents after partners' ADRD onset. Even larger impacts were observed for women and for non-Hispanic whites. Increased amounts of caregiving were associated with increases in depressive symptoms after a partner's ADRD onset. In all, ADRD has substantial impacts on family. Improved support mechanisms, including enriched community resources, clinician focus on dyadic needs, respite care, and policy efforts such as tax credits for caregivers, will be needed to meet the needs of couples increasingly affected by ADRD.

